# Replication and Virulence in Cattle of the First Lumpy Skin Disease Virus Isolated in Xinjiang, China (2019)

**DOI:** 10.1155/tbed/2315442

**Published:** 2025-07-01

**Authors:** Minmin Zhang, Shaohan Li, Yujie Shi, Xiaolong Xu, Zhiyuan Wen, Jinying Ge, Qiwei Zhang, Xinyin Lu, Xin Yin, Zhigao Bu

**Affiliations:** ^1^State Key Laboratory for Animal Disease Control and Prevention, Harbin Veterinary Research Institute, CAAS, Harbin, Heilongjiang Province, China; ^2^Key Laboratory of Zoonosis Prevention and Control of Guangdong Province, College of Veterinary Medicine, South China Agricultural University, Guangzhou, Guangdong Province, China

## Abstract

Before 2012, lumpy skin disease (LSD) was primarily confined to African countries. However, it rapidly spread to the Middle East and Southeast Europe, reaching the Balkans, the Caucasus, and the Russian Federation by 2015. The first confirmed case in China was reported on August 10, 2019, in Yili, Xinjiang, and on August 26, the Chinese government officially notified the World Organization for Animal Health (WOAH) of the outbreak. In this study, we isolated a LSD virus (LSDV) strain from a severely affected cattle skin sample collected in the Yili region of Xinjiang and designated it as strain Xinjiang/2019. Next-generation sequencing (NGS) analysis revealed that strain Xinjiang/2019 shared the highest similarity with the LSDV/Russia/Saratov/2017 strain based on full-length LSDV sequences available in NCBI. Phylogenetic analysis revealed that the Xinjiang/2019 isolate clustered with strains from China, Vietnam, Thailand, and Indonesia, forming a distinct phylogenetic branch. Recombination analysis further indicated that Xinjiang/2019 is predominantly a recombinant strain, derived from South African and European strains. To assess pathogenicity, cattle were infected with strain Xinjiang/2019 via intravenous (Group I) or intradermal (Group II) injection. In Group I, four out of five animals developed typical LSD symptoms, including fever (day 7), secondary nodules (day 8), rhinitis, conjunctivitis, and lymph node enlargement. Group II showed viremia by day 5 with milder symptoms. These findings indicate that strain Xinjiang/2019 is a virulent strain responsible for the first LSD outbreak in China.

## 1. Introduction

Lumpy skin disease (LSD) is a high-impact transboundary poxvirus disease affecting cattle and Asian water buffalo, caused by the LSD virus (LSDV). The World Organization for Animal Health (WOAH) lists LSD as a notifiable disease due to its rapid spread and significant economic impact on cattle production. LSDV is a DNA virus from the *Poxviridae* family, *Chordopoxvirinae* subfamily, and *Capripoxvirus* genus, which also includes goatpox and sheeppox viruses [1]. Cross-protection can occur among these three viruses, but serological differentiation is impossible. LSDV primarily transmits through mechanical vectors, mainly arthropods, though direct transmission between animals is less efficient [1, 2].

LSDV has a double-stranded DNA genome of approximately 151 kbp, with a central coding region flanked by 2.4 kbp inverted terminal repeats [3]. Bioinformatics analysis has predicted that LSDV approximately encodes 156 putative proteins [4]. Transmission electron microscopy reveals that LSDV virions have a biconcave core with a triple-coiled genomic structure, surrounded by two lateral bodies within the capsid [5].

The clinical severity of LSD varies based on virus virulence and host factors, such as immunity, age, and breed. Morbidity ranges from 3% to 85%, while mortality is typically under 10% [6], though it can occasionally rise as high as 75% during severe outbreaks [7]. Experimental studies show that the route of inoculation affects disease severity: intravenous injection of LSDV in cattle often leads to generalized disease, whereas intradermal inoculation results in more localized symptoms [8].

LSD was first identified in Zambia in 1929 and subsequently spread to the Middle East, Europe, and Asia [6, 9]. In China, the first confirmed outbreak occurred in Yili, Xinjiang, on August 10, 2019, and was officially reported to WOAH later that month.

In this study, we report the first isolation and characterization of a LSDV strain from China, designated Xinjiang/2019. The isolate was identified using PCR, immunofluorescence, western blotting, electron microscopy, and full-genome sequencing. Phylogenetic analysis was conducted to evaluate its relationship to other LSDV strains. We also assessed the virus's pathogenicity in cattle following experimental infection via different inoculation routes. Our findings provide key insights into the molecular and clinical features of the strain responsible for China's first LSD outbreak.

## 2. Materials and Methods

### 2.1. Facility and Ethics Statements

All live LSDV experiments were conducted in enhanced Biosafety Level 3 (BSL-3) facilities at the Harbin Veterinary Research Institute (HVRI), Chinese Academy of Agricultural Sciences (CAAS), with approval from the Ministry of Agriculture and Rural Affairs. All animal procedures were approved by the Animal Ethics Committee of HVRI under protocol number (HVRISQ-23-46). The study strictly followed the guidelines in the Guide for the Care and Use of Laboratory Animals issued by the Ministry of Science and Technology of the People's Republic of China.

### 2.2. Cell Culture and Virus Isolation

Primary lamb testis (LT) cells were cultured in Eagle's Minimum Essential Medium (MEM) (Hyclone, USA) supplemented with 10% fetal bovine serum (FBS) and 2% penicillin-streptomycin, at 37°C in a 5% CO_2_ incubator. Skin samples from symptomatic cattle were excised into 2–3 cm pieces, ground with liquid nitrogen, and homogenized in 1x PBS containing 10% penicillin-streptomycin. The resulting homogenate was used to inoculate LT cells for virus isolation. Infected cells were harvested when approximately 70%–80% cytopathic effects (CPEs) were observed and stored at −70°C.

### 2.3. PCR and Quantitative PCR (qPCR)

LSDV genomic DNA was extracted from skin samples using the TIANamp Genomic DNA Kit (TIANGEN, China). PCR primers and amplification conditions were applied according to the WOAH-recommended protocol as follows:

Forward primer 5′-TCC-GAG-CTC-TTT-CCT-GAT-TTT-TCT-TAC-TAT-3′

Reverse primer 5′-TAT-GGT-ACC-TAA-ATT-ATA-TAC-GTA-AAT-AAC-3′ [10].

The PCR reaction was performed in a final volume of 50 μL consisting of 25 μL of 2× PCR buffer, 1.5 μL of MgCl_2_ (50 mM), 1 μL of dNTP mix (10 mM), 1 μL of forward primer, 1 μL of reverse primer, 1 μL of DNA template (~10 ng), 0.5 μL of Taq DNA polymerase, and nuclease-free water adjusted to maintain the final volume. The volume of the DNA template may vary, and the amount of nuclease-free water should be adjusted accordingly to maintain a final volume of 50 μL.

LSDV genomic DNA was extracted using the TIANamp Genomic DNA Kit (TIANGEN, China) from tissue homogenates, as well as oral, nasal, and ocular swabs, or EDTA-treated whole blood. qPCR was performed on a QuantStudio 5 system (Applied Biosystems, USA), following the protocol described by Alexander et al. [11]. The primers and probe used in the assay were as follows: Forward primer, 5′-CCTCCTTTTAAGCTACTTTTTCTTA-3′; reverse primer, 5′-GATACATGTAGGAACATTGTTACCTA-3′; and probe, 5′-FAM-ACCACCTAATGATAG-BHQ1-3′. Specifically, quantification was performed using a standard curve generated from 10-fold serial dilutions of a plasmid containing the target gene sequence, ranging from 10^7^ to 10^1^ copies/μL. Each dilution point on the standard curve was run in triplicate. The qPCR reaction efficiency was 98.2%, with an *R*^2^ value of 0.998. The limit of detection (LOD) was determined to be 10 copies per reaction, based on repeated testing of low-concentration standards with a ≥95% detection rate.

### 2.4. Immunofluorescence Assay (IFA)

For the indirect IFA, LT cells were seeded in 24-well plates and infected with LSDV Xinjiang at a multiplicity of infection (MOI) of 0.05. About 72 h postinfection, the cells were fixed with 3% paraformaldehyde for 15 min at room temperature, permeabilized with 0.1% (*w*/*v*) Triton X-100 for 10 min, and then incubated with cattle LSDV-positive serum as the primary antibody. Fluorescein isothiocyanate (FITC)-conjugated goat anticattle IgG was used as the secondary antibody. Cell nuclei were stained with DAPI at room temperature for 1 h, followed by three washes with PBS. The FITC fluorescent signal was visualized using a fluorescence microscope (Axio Observer.Z1; Zeiss).

### 2.5. Electron Microscopy

In this study, negative staining of whole-mount viral particle and thin sectioning of resin-embedded cells with viruses were employed. Cell supernatants containing LSDV particles were collected, centrifuged, and fixed with 2.5% glutaraldehyde (pH 7.2). A 400-mesh hexagonal electron microscope grid, precoated with pioloform-carbon substrate activated by glow discharge in pentylamine vapor, was floated onto a drop of the suspension placed on parafilm or a wax plate. After 1 min, the grid was transferred to a drop of Tris/EDTA buffer (pH 7.8) for 20 s, followed by a drop of 2% phosphotungstic acid (pH 7.2) for 10 s (http://www.oie.int/). LT cells infected with LSDV were washed with PBS, fixed with 2.5% glutaraldehyde (pH 7.2) at 4°C overnight, and postfixed with 1% OsO_4_ (pH 7.4) at 4°C for 2 h. The cells were then dehydrated in stepwise acetone at 4°C and embedded in 812 Epon resin. Thin sections were stained with 1% uranyl acetate (pH 6.5) and 1% lead citrate (pH 7.2). The grid was examined using an H-7650 electron microscope (Hitachi, Tokyo, Japan) operating at 80 kV.

### 2.6. Virus Growth Titration

LT cells were infected with the LSDV isolate at a MOI of 0.3. Cell supernatants and cells were collected at various time points postinfection. First, the cell supernatants were collected, followed by washing the cell monolayers three times with PBS. Equal volumes of PBS were then added to the monolayers. All samples were stored at −70°C, subjected to three freeze–thaw cycles, and subsequently inoculated onto LT cells to determine viral titers by observing the CPE.

### 2.7. Genetic Evolutionary Analysis

MEGA X software was used to construct the phylogenetic tree. Sequence alignments were analyzed using appropriate models of nucleotide substitution, and the phylogenetic tree was inferred using the neighbor-joining (NJ) method. Bootstrap values were calculated with 1000 replications to assess statistical support for tree branches. The phylogenetic tree was further optimized and visualized using the Interactive Tree of Life (iTOL) web server.

Recombination analysis was performed using RDP4 v4 software, employing the RDP, BootScan, GENECOV, Maxchi, Chimera, SiScan, and 3Seq algorithms. Recombination events were visualized through a similarity plot, with the Xinjiang/2019 strain designated as the reference strain. The similarity plot was generated and analyzed using SimPlot v. 3.5.1 software, applying the two-parameter (Kimura) model, with a sliding window of 1000 bp and a step size of 30 bp.

### 2.8. Animal Experiments

Animal experiments were conducted in the animal BSL-3 facilities at HVRI, following a protocol approved by the Animal Ethics Committee of HVRI, CAAS, and Heilongjiang Province, China. In total 13 one- to one-and-a-half-year-old male Chinese yellow cattle, seronegative for LSDV, were randomly assigned to three groups. Groups I and II each consisted of five cattle, which were infected with LSDV Xinjiang/2019 via intravenous injection and intradermal inoculation, respectively, at a dose of 10^6.5^ TCID_50_. Group III consisted of three cattle mock-infected with PBS as the control. Each group was housed separately to prevent cross-contamination. The animals were monitored daily for abnormalities, and their rectal temperatures were measured up to 21 days postinfection (dpi). They were euthanized at the end of the experiment or when necessary. Whole blood (with and without EDTA) was collected daily for virus detection. Nasal, oral, and ocular swabs were taken from days 1 to 21 pi at 2-day intervals. Samples were placed in tubes containing 2 mL DMEM with penicillin G (500 IU) and streptomycin sulfate (500 µg), then stored at −70°C until further use.

### 2.9. ELISA Assay

A commercial ELISA kit (Capripox/LSD Ab. ELISA Kit, KERNEL, USA) was used to detect specific antibodies against LSDV. The testing was performed according to the manufacturer's instructions. Results were determined based on the calculated sample-to-positive (S/P) ratio. If the S/P ratio exceeded 40%, the sample was considered positive for antibodies against LSDV. The formula for calculating the S/P ratio is as follows:

  $$\begin{array}{c} S/P=\frac{\text{Test sample OD}-\text{negtive control OD}}{\text{Positive control OD}-\text{negtive control OD}}\times \;100. \end{array}$$

The serum neutralization (SN) antibody titer was determined using a conventional method [12]. Virus isolation from tissues was performed on LT cells cultured in 12-well culture plates. CPEs were observed 5 days after inoculation of the sample supernatant.

## 3. Results

### 3.1. Isolation and Characterization of LSDV Isolate

**Figure 1:**
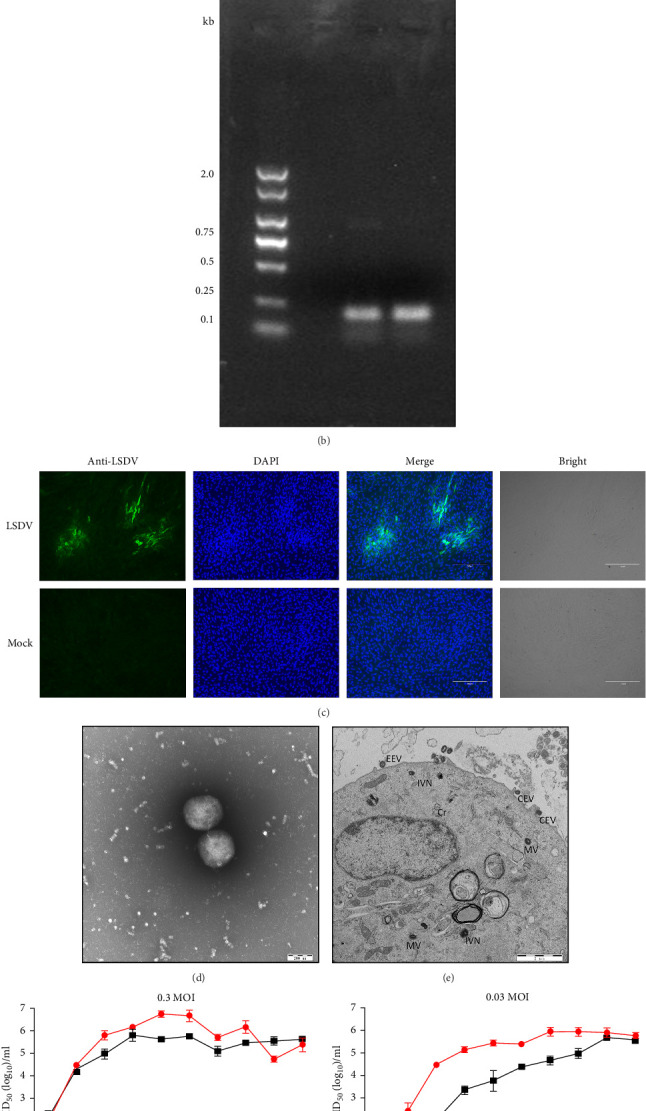
Identification of an LSDV strain from a field sample of skin tissue. (A) LT cells exhibited cytopathic effects (CPEs) following inoculation with homogenized skin sample supernatant. CPE was observed at 6 days postinfection (dpi). (B) PCR detection of total DNA from the skin sample. Line 1, negative control; lane 2, positive control; lane 3, sample. (C) Identification the virus using of an immunofluorescence assay with cells infected with sample. (D) Electron microscopy of negatively stained viral particles. The supernatant from infected LT cells was collected, concentrated and negative staining electron microscopy was performed. Spherical, enveloped mature particles were observed. (E) Electron microscopy of thin sections from infected LT cells. After 96 h postinfection, various poxvirus particle forms were observed, including crescent-shaped particles (Cr), immature virus (IV), immature virus with nucleoids (IVN), and mature virus (MV). Many MVs were wrapped by intracellular membranes derived from the Trans-Golgi network, forming intracellular enveloped virus (IEV). (F) and (G) Growth curve of LSDV Xinjiang/2019. LT cells were infected with LSDV Xinjiang/2019 at an MOI of 0.03 or 0.3. Cells or cell culture supernatant were, respectively, harvested at different times by freeze–thawing three times. The supernatants were then centrifuged, and viral titers were determined by inoculating LT cells.

A skin sample from cattle with typical clinical symptoms was inoculated onto LT cells. CPEs were observed on day 6 postinoculation (Figure 1A). The first passage stock was generated on day 9, yielding a titer of 10^6.25^ TCID_50_/mL. PCR targeting the LSDV *P32* gene confirmed the presence of LSDV in the cell supernatants on day 6, with a positive 192-bp PCR product detected (Figure 1B). Furthermore, an indirect IFA using positive LSDV serum showed green fluorescence at the sites of CPEs (Figure 1C). Electron microscopy revealed typical poxvirus morphology and four types of virus particles: Intracellular mature virus (IMV), intracellular enveloped virus (IEV), cell-associated enveloped virus (CEV), and extracellular enveloped virus (EEV), along with immature virus particles (IV) and crescent-shaped IV with nucleoids (IVN) (Figure 1D,E).

To assess viral growth dynamics, LT cells were infected at multiplicities of infection of 0.03 and 0.3. Given that poxviruses produce two distinct virions: Intracellular virus (IV) and extracellular virus (EV) [13], cell supernatants and cells were collected at different time points postinfection for virus titration. At MOI 0.03, intracellular virus titers were higher than EV titers, reaching a peak of 10^5.92^TCID_50_/mL on day 7. At MOI 0.3, EV reached a peak titer of 10^6.79^ TCID_50_/mL on day 5 (Figure 1F,G). These results confirmed the successful isolation of LSDV from the field outbreak sample, designated LSDV Xinjiang/2019.

### 3.2. Sequence Analysis of the LSDV Xinjiang/2019 Isolate

**Figure 2:**
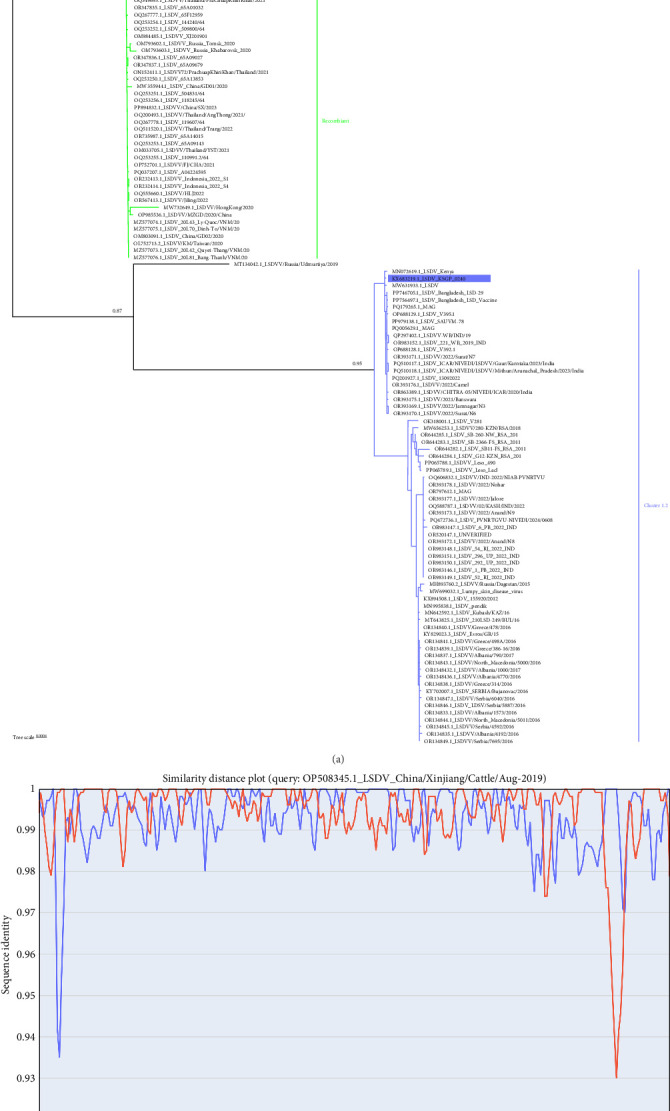
Evolutionary analyses of genetic recombination. (A) Phylogenetic analysis of the LSDV Xinjiang/2019 strain based on its complete genome and recombination analyses. Viral genome sequences of representative LSDV strains were retrieved from the NCBI database. Phylogenetic trees were constructed using the neighbor-Joining method in MEGA X software (https://www.megasoftware.net/). In the phylogenetic tree, the orange branches represent Cluster 1.1, the green branches indicate recombinant branches, and the blue branches correspond to Cluster 1.2. (B) Analysis of the recombinant OP508345.1 (LSDV Xinjiang/2019 strain), resulting from recombination between the KX683219.1 (LSDV KSGP 0240) and AF409138.1 (LSDV Neethling vaccine LW 1959) strains. The *x*-axis and *y*-axis represent the genomic nucleotide position and genetic similarity, respectively.

Full-length genome sequencing, excluding the terminal regions, revealed a nucleotide composition of 74.2% A + T and 25.8% C + G, in agreement with previous reports [14]. Tracing forward in time, comparative analysis with available LSDV genomes indicated that the Xinjiang/2019 strain shared a high degree of identity with LSDV/Russia/Saratov/2017, a recombinant strain derived from both a vaccine and a wild-type virus. Phylogenetic analysis clustered the Xinjiang/2019 isolate with strains from China, Vietnam, Thailand, and Indonesia in a distinct recombinant cluster, suggesting a relationship with vaccine-derived strains (Figure 2A). These findings support the hypothesis that the Xinjiang/2019 isolate likely originated from a vaccine-related strain. Recombination events at the whole-genome level were analyzed for 132 LSDV strains, including Xinjiang/2019 strain. Overall, the Xinjiang/2019 strain is primarily a recombinant derived from Cluster1.1 and Cluster1.2 strains (Figure 2B).

### 3.3. Pathogenicity of LSDV Xinjiang/2019 in Cattle

**Figure 3:**
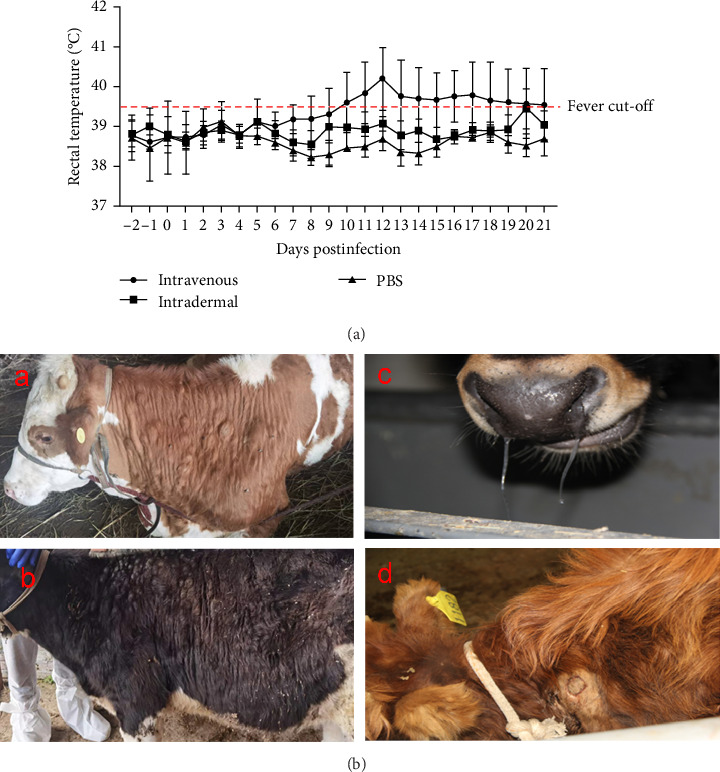
Temperature variations following viral infection in different experimental groups. (A) Temperature changes in response to varying viral infection doses. Data are presented as mean values with standard deviations for each group. The red dotted line indicates the fever threshold. (B) Systemic skin nodules appeared from day 9 postinfection (dpi) in both Group I and Group II (a, b). Salivation and rhinitis were observed beginning on dpi 11 (c). (d) Swelling at the injection site was observed in Group I.

**Figure 4:**
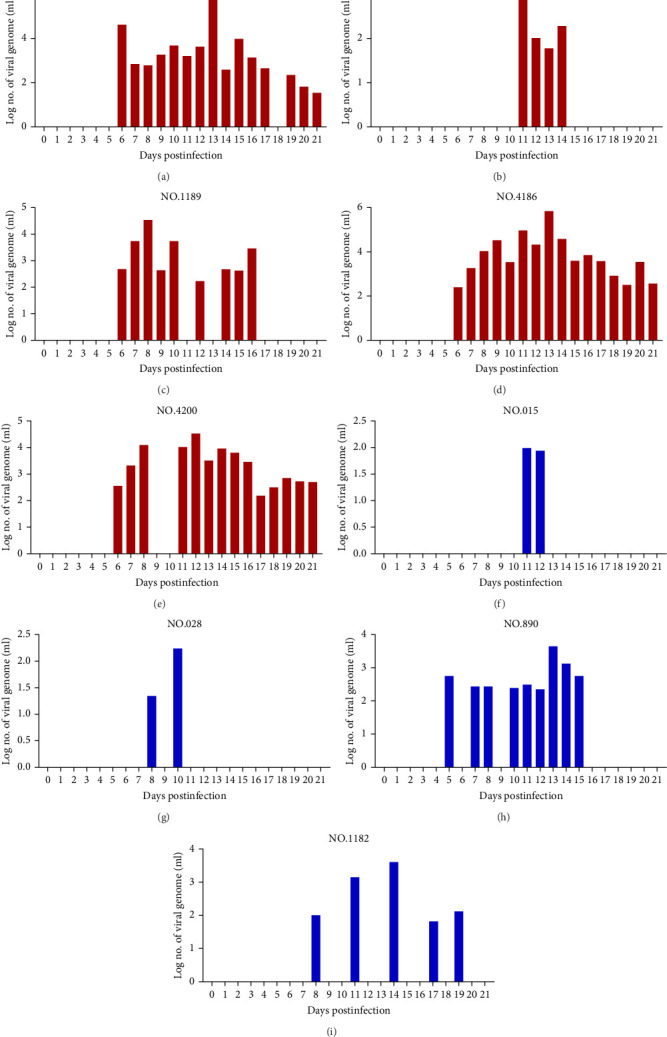
Viral genomic copies in samples collected from Groups I and II. Whole blood samples were collected at the indicated time points from Group I (A–E) and Group II (F–I). Viral genome copies were quantified using a qPCR assay.

Cattle were divided into three groups for infection, as described in Table 1. Four out of five cattle (no. 879, no. 1188, no. 4186, and no. 4200) in the intravenous inoculation group developed fever (>39.5°C) between days 10 and 21 postinoculation (Figure 3A), with three (no. 879, no. 4186, and no. 4200) exhibiting severe clinical symptoms typical of LSD, including secondary nodules, from days 6 to 21 postinoculation (Figure 3A,B). Viremia and viral shedding were observed starting from day 6 postinfection (Figure 4A–E and Table 2). Secondary nodules appeared on day 8.

In Group II, with intradermal inoculation, cattle exhibited mild symptoms with no fever and only two out of five cattle (no. 890 and no. 1182) developing secondary nodules by day 10 postinoculation. The injection site in one animal (no. 890) swelled and ruptured by day 7. Viremia was present from days 5 to 19, with viral DNA detected in blood and swabs from natural orifices (Figure 4F–I; Table 2).

At the end of the experiment, all the cattle were euthanized, and prespecified tissues were collected for viral DNA detection and virus isolation. Consistent with the clinical symptoms, viral DNA, and virus isolation were observed in all tissues from cattle no. 4186 and no. 4200 in Group I (Table 3). For cattle no. 879, viral DNA was detected, and virus isolation was successful in most tissues, except for the lymph node and peripheral blood (Table 3). Viral DNA and virus isolation were also positive at the injection site and secondary nodules in cattle no. 1188. In contrast, only viral DNA was detected at the injection site of cattle no. 889 (Table 3). In Group II, viral genomic DNA was detected at lower levels in cattle no. 031, no. 028, and no. 015, and no viral nucleic acid was detected in samples from other tissues except the injection site (Table 3). Virus isolation results were consistent with the qPCR findings. Notably, viruses were more readily isolated from cattle exhibiting typical LSD symptoms.

### 3.4. Adaptive Humoral Immune Responses to Xinjiang/2019

ELISA and virus neutralization assays (VNA) were used to assess antibody responses to LSDV. Total antibodies were first detected in group I cattle at 14 dpi, with the highest titers reached at dpi 21 in cattle no. 879 and no. 1188 (Table 4). In Group II, antibodies were detectable only at dpi 21, with comparable titers in cattle no. 890 and no. 1182 (Table 4). Group I exhibited a higher seroconversion rate of LSDV IgG antibodies than Group II (Table 5).

**Figure 5:**
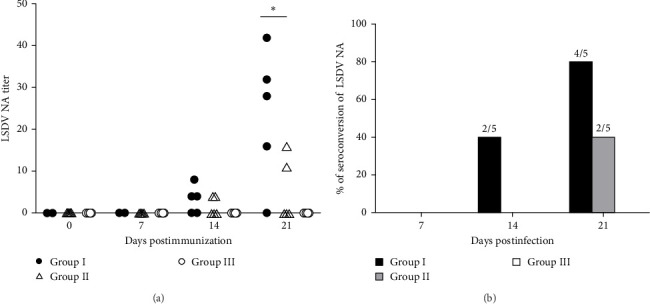
Titers and seroconversion rates of neutralizing antibodies (NAs) against LSDV. Serum samples collected at specific time points during the study were analyzed using the serum neutralization test (SNT). (A) neutralizing antibody (NA) titers against LSDV were determined in the collected sera. Each point represents the NA titer of an individual cattle. (B) The seroconversion rates of NAs against LSDV were evaluated. Statistical significance was assessed using the *t*-test (*⁣*^*∗*^, *p*  < 0.05; *⁣*^*∗∗*^, *p*  < 0.01).

Serum neutralizing antibodies were detected starting at 14 dpi in cattle no. 1188, no. 4200, and no. 879 in Group I, with the highest titers reaching 1:42 at dpi 21 (Figure 5A). Four out of five cattle in Group I produced SN antibodies, while only two out of five cattle in Group II showed SN activity, with titers up to 1:16 (Figure 5B). These results suggest a robust immune response in cattle with severe clinical symptoms.

## 4. Discussion

This study presents the first successful isolation and characterization of LSDV from a field outbreak in Xinjiang, China. Genomic and pathogenicity analyses of strain Xinjiang/2019 confirmed its virulence and ability to replicate in cattle. Phylogenetic analysis revealed that Xinjiang/2019 shares high sequence similarity with strains previously identified in Russia, including LSDV/Kurgan/2018 (GenBank: OR194148.1) and LSDV/Russia/Saratov/2019 (OM530217.1), LSDV/Russia/Tyumen/2019 (OL542833.1) and LSDV/Russia/Saratov/2017 (MH646674.1) [14]. These Russian strains, known to cause typical LSD-like symptoms, are considered recombinant viruses derived from both vaccine and wild-type strains [15]. Specifically, the genome of Xinjiang/2019 harbors 27 recombination events with an average distance of 2.4 kb between breakpoints [16]. This recombinant lineage has been repeatedly detected in subsequent outbreaks in mainland China, Hong Kong, Taiwan, Vietnam, Thailand, and Indonesia [17, 18], suggesting regional spread. In contrast, strains circulating in India and Bangladesh appear to have evolved through a separate lineage [18]. The presence of a recombinant genome in Xinjiang/2019 underscores the need for continued genomic surveillance and evolutionary analysis to guide LSDV control and vaccination strategies in affected regions [19].

Electron microscopy confirmed the presence of multiple forms of virions during replication, consistent with previous studies on poxvirus [20, 21]. The isolate demonstrated efficient replication in LT cells, reaching a peak intracellular virus titer of 10^6.79^ TCID_50_/mL at an MOI of 0.3 on day 5 postinfection. This finding aligns with prior research indicating that IMV is the most abundant virion during LSDV infection [21].

Less than 50% of cattle experimentally infected with LSDV or naturally exposed during an outbreak typically develop generalized infection [8, 22, 23]. In this study, we used two common administration routes: Intravenous and intradermal inoculation. However, intravenous injection consistently reproduced acute LSD in cattle, which is the route, that is, most likely to induce generalized disease [8]. The recorded incubation period of 5–10 days in the cattle aligns with previous reports, where the incubation period following experimental infection ranged from 4 to 14 days [22, 24–26]. The clinical signs observed were consistent with those in earlier descriptions of experimentally induced LSD, varying from mild cases with a few secondary skin nodules to more severe, generalized infections, depending on the route of inoculation [8, 26, 27]. A correlation between viral shedding and clinical symptoms was also observed.

Serological analysis detected total antibodies against LSDV via ELISA starting from day 14 postinfection, and neutralizing antibodies (NAs) followed a similar pattern, confirming successful viral colonization. Disease severity appeared to be influenced by factors such as the animal's health, immune status, breed, and viral strain. These findings highlight the importance of considering inoculation routes when developing vaccination strategies.

## 5. Conclusions

This study successfully isolated and characterized a virulent LSDV strain, advancing our understanding of its biological properties. The research highlights the role of virulence and administration route in disease progression, offering crucial insights for LSDV control through early detection, vaccination, and optimized inoculation methods.

## Figures and Tables

**Table 1:** The group setting for animal experiment.

Group	Animal no.	Dose	Inoculum
Group I	879	1 × 10^6^ TCID_50_/mL	Intravenous
	889		
	1188		
	4186		
	4200		

Group II	015	1 × 10^6^ TCID_50_/mL	Intradermal
	028		
	031		
	890		
	1182		

Group III	600	PBS	Intradermal
	1181		
	4185		

**Table 2:** The viral shedding in swabs of cattle inoculated with LSDV.

Group	Animal no.	Swab site	1	3	5	7	9	11	13	15	17	19	21(dpi)
Group I	879	Ocular	0.00	0.00	2.00	2.05	1.98	2.68	2.09	2.14	2.77	3.18	2.59
		Nasal	0.00	0.00	1.98	1.85	2.78	4.07	6.01	5.42	4.34	4.59	3.79
		Oral	0.00	0.00	0.00	0.00	1.78	3.62	3.80	2.62	3.11	2.66	2.34
	889	Ocular	0.00	0.00	0.00	0.00	1.84	2.66	2.70	2.15	3.37	2.98	2.50
		Nasal	0.00	0.00	1.78	2.21	3.32	3.09	0.00	0.00	2.55	2.68	2.09
		Oral	0.00	0.00	0.00	0.00	2.53	2.68	2.36	0.00	0.00	1.78	2.14
	1188	Ocular	0.00	0.00	2.00	1.94	2.21	2.24	2.24	1.89	2.59	2.65	2.18
		Nasal	0.00	0.00	0.00	0.00	0.00	1.75	1.85	0.00	0.00	2.58	0.00
		Oral	0.00	0.00	0.00	0.00	1.89	1.91	2.79	0.00	2.11	2.28	0.00
	4186	Ocular	0.00	0.00	0.00	0.00	4.28	2.29	2.57	2.09	3.28	4.16	3.94
		Nasal	0.00	0.00	0.00	2.45	3.92	4.78	4.70	3.86	3.91	3.94	5.26
		Oral	0.00	0.00	1.86	2.19	3.16	3.75	3.25	2.20	2.60	2.97	4.38
	4200	Ocular	0.00	0.00	2.00	1.94	2.21	2.24	2.24	1.89	2.59	2.65	2.18
		Nasal	0.00	0.00	0.00	0.00	0.00	1.75	1.85	0.00	0.00	2.58	0.00
		Oral	0.00	0.00	0.00	0.00	1.89	1.91	2.79	0.00	2.11	2.28	0.00

Group II	015	Ocular	0.00	0.00	0.00	0.00	0.00	1.80	0.00	0.00	1.78	2.27	2.47
		Nasal	0.00	0.00	0.00	0.00	0.00	0.00	0.00	0.00	0.00	2.31	0.00
		Oral	0.00	0.00	0.00	0.00	0.00	0.00	0.00	0.00	0.00	0.00	1.95
	028	Ocular	0.00	0.00	0.00	0.00	0.00	1.80	2.42	1.74	3.08	3.59	3.14
		Nasal	0.00	0.00	0.00	1.26	0.00	1.99	2.36	0.00	2.00	2.03	1.98
		Oral	0.00	0.00	0.00	0.00	0.00	0.00	1.85	0.00	0.00	0.00	2.35
	031	Ocular	0.00	0.00	0.00	0.00	0.00	0.00	0.00	2.38	0.00	0.00	0.00
		Nasal	0.00	0.00	0.00	0.00	0.00	1.76	0.00	0.00	0.00	0.00	1.86
		Oral	0.00	0.00	0.00	0.00	0.00	0.00	1.83	0.00	0.00	0.00	0.00
	890	Ocular	0.00	0.00	0.00	1.82	3.12	4.39	2.71	2.69	2.90	2.14	2.76
		Nasal	0.00	0.00	0.00	2.26	3.67	4.41	4.92	4.81	6.54	4.51	4.51
		Oral	0.00	0.00	0.00	1.78	2.69	3.68	3.52	3.15	4.08	2.84	2.44
	1182	Ocular	0.00	0.00	2.23	2.67	2.08	1.84	2.66	0.00	2.97	0.00	1.89
		Nasal	0.00	0.00	1.67	1.02	2.57	4.54	3.94	3.60	4.24	2.80	3.52
		Oral	0.00	0.00	0.00	0.00	1.69	3.02	3.07	0.00	1.90	2.24	3.65

*Note:* Viral copies are expressed as a logarithmic (base 10) copies per milliliter (log_10_copies/mL).

**Table 3:** The results for qPCR and virus isolation.

Group	Animal no.	Lymoph node		Injection site		Secondary nodule		Peripheral blood	
		qPCR	Virus isolation	qPCR	Virus isolation	qPCR	Virus isolation	qPCR	Virus isolation
I	879	+	−	+	+	+	+	+	−
	889	−	−	+	−	/	/	−	−
	1188	−	−	+	+	+	+	−	−
	4186	+	+	+	+	+	+	+	+
	4200	+	+	+	+	+	+	+	+

II	015	−	−	+	−	/	/	−	−
	028	−	−	+	−	/	/	−	−
	031	−	−	+	−	/	/	−	−
	890	+	+	+	+	+	−	+	−
	1182	+	+	+	+	+	−	+	+

*Note:* (+): Positive result. (−): Negative result. (/): Not performed.

**Table 4:** The total antibodies measured by ELISA.

Group	Animal no.	Dpi 0	Dpi 7	Dpi 14	Dpi 21
		S/P	S/P	S/P	S/P
I	4186	0.47	5.23	26.98	61.18
	1188	5.33	10.45	40.54	95.53
	4200	0.63	6.50	36.44	113.57
	879	2.04	2.40	194.21	341.49
	889	1.25	3.53	10.88	31.53

II	890	3.92	3.95	18.79	40.18
	1182	4.39	4.38	34.89	46.27
	028	2.84	6.92	15.11	8.16
	015	5.95	12.15	9.60	6.27
	031	−2.82	2.54	4.24	5.08

III	600	2.47	6.92	6.07	4.24
	1181	1.89	7.20	6.64	6.43
	4185	2.08	3.81	2.68	1.88

**Table 5:** Rate of seroconversion of LSDV IgG antibody.

Group	Dpi 0	Dpi 7	Dpi 14	Dpi 21
Group I	0/5	0/5	2/5	4/5
Group II	0/5	0/5	0/5	2/5
Group III	0/3	0/3	0/3	0/3

## Data Availability

The datasets used and/or analyzed during the current study are available from the corresponding author upon reasonable request.
